# Impact of industrial robots on environmental pollution: evidence from China

**DOI:** 10.1038/s41598-023-47380-6

**Published:** 2023-11-26

**Authors:** Yanfang Liu

**Affiliations:** Harbin Vocational College of Science and Technology, Harbin, 150300 Heilongjiang People’s Republic of China

**Keywords:** Ecology, Environmental sciences, Environmental social sciences

## Abstract

The application of industrial robots is considered a significant factor affecting environmental pollution. Selecting industrial wastewater discharge, industrial SO_2_ emissions and industrial soot emissions as the evaluation indicators of environmental pollution, this paper uses the panel data model and mediation effect model to empirically examine the impact of industrial robots on environmental pollution and its mechanisms. The conclusions are as follows: (1) Industrial robots can significantly reduce environmental pollution. (2) Industrial robots can reduce environmental pollution by improving the level of green technology innovation and optimizing the structure of employment skills. (3) With the increase in emissions of industrial wastewater, industrial SO_2_, and industrial dust, the impacts generated by industrial robots are exhibiting trends of a “W” shape, gradual intensification, and progressive weakening. (4) Regarding regional heterogeneity, industrial robots in the eastern region have the greatest negative impact on environmental pollution, followed by the central region, and the western region has the least negative impact on environmental pollution. Regarding time heterogeneity, the emission reduction effect of industrial robots after 2013 is greater than that before 2013. Based on the above conclusions, this paper suggests that the Chinese government and enterprises should increase investment in the robot industry. Using industrial robots to drive innovation in green technology and optimize employment skill structures, reducing environmental pollution.

## Introduction

Since the reform and opening up, China’s rapid economic growth has created a world-renowned “economic growth miracle”^1^. With the rapid economic growth, China’s environmental pollution problem is becoming more and more serious^2^. According to the “*Global Environmental Performance Index Report*” released by Yale University in the United States in 2022, China’s environmental performance index scores 28.4 points, ranking 160th out of 180 participating countries. The aggravation of environmental pollution not only affects residents’ health^3^, but also affects the efficiency of economic operation^4^. According to calculation of the General Administration of Environmental Protection, the World Bank and the Chinese Academy of Sciences, China’s annual losses caused by environmental pollution account for about 10% of GDP. Exploring the factors that affect environmental pollution and seeking ways to reduce environmental pollution are conducive to the development of economy within the scope of environment.

**Figure 1:**
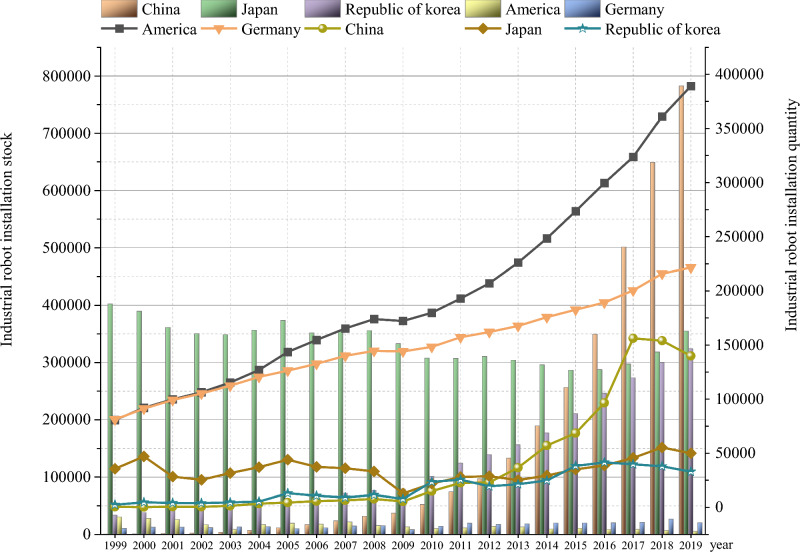
Industrial robot installations in the world’s top five industrial robot markets from 1999 to 2019.

Industrial robots are machines that can be automatically controlled, repeatedly programmed, and multi-purpose^5^. They replace the low-skilled labor force engaged in procedural work^6^, reducing the raw materials required for manual operation. Industrial robots improve the clean technology level and energy efficiency of coal combustion, reducing pollutant emissions in front-end production. Industrial robots also monitor the energy consumption and sewage discharge in the production process in real time. The excessive discharge behavior of enterprises in the production process is regulated, reducing the emission of pollutants in the end treatment. Based on the selection and coding of literature (Appendix A), this paper uses the meta-analysis method to compare the impacts of multiple factors such as economics, population, technology, and policy on environmental pollution. As shown in Table 1, compared to other factors, industrial robots demonstrate greater advantages in reducing environmental pollution. There is a lack of research on the relationship between industrial robots and environmental pollution in China. With the advent of artificial intelligence era, China’s industrial robot industry has developed rapidly. According to data released by the International Federation of Robotics (IFR), from 1999 to 2019, China’s industrial robot ownership and installation shows an increasing trend year by year (Fig. 1). In 2013 and 2016, China’s industrial robot installation (36,560) and ownership (349,470) exceeds Japan for the first time, becoming the world’s largest country in terms of installation and ownership of industrial robots. Whether the application of industrial robots in China contributes to the reduction of environmental pollution? What is the mechanism of the impact of China’s industrial robots on environmental pollution? Researching this issue is crucial for filling the gaps in existing research and providing a reference for other countries to achieve emission reduction driven by robots.

**Table 1:** The impact of various factors on environmental pollution.

Classification	N	r	Two-tailed test		95% confidence interval	
			Z value	*P* value	Low	High
Economic factor	3800	− 0.008	− 0.776	0.438	− 0.026	0.011
Demographic factor	4041	− 0.082	0.542	0.588	− 0.361	0.211
Technological factor	49,100	− 0.130	− 1.199	0.230	− 0.332	0.083
Policy factor	3252	− 0.128	− 2.539	0.011	− 0.360	− 0.048
Industrial robots	1944	0.201	8.978	0.000	0.158	0.243

Based on the above analysis, this paper innovatively incorporates industrial robots and environmental pollution into a unified framework. Based on the panel data of 30 provinces in China from 2006 to 2019, this paper uses the ordinary panel model and mediating effect model to empirically test the impact of industrial robots on China’s environmental pollution and its transmission channels. The panel quantile model is used to empirically analyze the heterogeneous impact of industrial robots on environmental pollution under different environmental pollution levels.

## Literature review

A large number of scholars have begun to study the problem of environmental pollution. Its research content mainly includes two aspects: The measurement of environmental pollution and its influencing factors. Regarding the measurement, some scholars have used SO_2_ emissions^7^, industrial soot emissions^8^ and PM2.5 concentration^9^ and other single indicators to measure the degree of environmental pollution. The single indicator cannot fully and scientifically reflect the degree of environmental pollution. To make up for this defect, some scholars have included industrial SO_2_ emissions, industrial wastewater discharge and industrial soot emissions into the environmental pollution evaluation system, and used the entropy method to measure environmental pollution level^10^. This method ignores the different characteristics and temporal and spatial trends of different pollutants, which makes the analysis one-sided. Regarding the influencing factors, economic factors such as economic development level^11^, foreign direct investment^12^ and income^13^, population factors such as population size^14^ and urbanization level^15^, energy consumption^16^ all have an impact on environmental pollution. Specifically, economic development and technological innovation can effectively reduce environmental pollution^17^. The expansion of population size can aggravate environmental pollution. Income inequality can reduce environmental pollution, but higher income inequality may aggravate environmental pollution^18^. There are “pollution heaven hypothesis” and “pollution halo hypothesis” between foreign direct investment and environmental pollution^19^. Technological factors also have a non-negligible impact on environmental pollution^20^.

With continuous deepening of research, scholars have begun to focus on the impact of automation technology, especially industrial robot technology, on the environment. Ghobakhloo et al.^21^ theoretically analyzed the impact of industrial robots on energy sustainability, contending that the application of industrial robots could foster sustainable development of energy. Using data from multiple countries, a few scholars have empirically analyzed the effect of industrial robots on environmental pollution (Table 2). Luan et al.^22^ used panel data from 73 countries between 1993 and 2019 to empirically analyze the impact of industrial robots on air pollution, finding that the use of industrial robots intensifies environmental pollution. Using panel data from 66 countries from 1993 to 2018, Wang et al.^23^ analyzed the impact of industrial robots on carbon intensity and found that industrial robots can reduce carbon intensity. On the basis of analyzing the overall impact of industrial robots on environmental pollution, some scholars conducted in-depth exploration of its mechanism. Based on data from 72 countries between 1993 and 2019, Chen et al.^5^ explored the impact of industrial robots on the ecological footprint, discovering that industrial robots can reduce the ecological footprint through time saving effect, green employment effect and energy upgrading effect. Using panel data from 35 countries between 1993 and 2017, Li et al.^24^ empirically examined the carbon emission reduction effect of industrial robots, finding that industrial robots can effectively reduce carbon emissions by increasing green total factor productivity and reducing energy intensity. Although the above studies have successfully estimated the overall impact of industrial robots on environmental pollution and its mechanisms, they have not fully considered the role of technological progress, labor structure and other factors in the relationship between the two. These studies all chose data from multiple countries as research samples and lack research on the relationship between industrial robots and environmental pollution in China, an emerging country.

**Table 2:** Review of the relationship between industrial robots and environmental pollution in recent years.

References	Sample	Explanatory variables	Explained variable	Effects	Mechanism
Ghobakhloo et al.^21^	No	No	No	Positive	No
Luan et al.^22^	73 countries	Industrial robots	Air environment	Negative	No
Chen et al.^5^	72 countries	Industrial robots	Ecological footprint	Negative	Time saving, green employment, energy upgrade
Li et al.^24^	35 countries	Industrial robots	Carbon emission	Negative	Green total factor productivity, energy intensity
Wang et al.^23^	66 countries	Industrial robots	Carbon intensity	Negative	No

The above literature provides inspiration for this study, but there are still shortcomings in the following aspects: Firstly, there is a lack of research on the relationship between industrial robots and environmental pollution in emerging countries. There are significant differences between emerging and developed countries in terms of institutional background and the degree of environmental pollution. As a representative emerging country, research on the relationship between industrial robots and environmental pollution in China can provide reliable references for other emerging countries. Secondly, theoretically, the study of the impact of industrial robots on environmental pollution is still in its initial stage. There are few studies that deeply explore its impact mechanism, and there is a lack of analysis of the role of technological progress and labor structure in the relationship between the two.

The innovations of this paper are as follows: (1) In terms of sample selection, this paper selects panel data from 30 provinces in China from 2006 to 2019 as research samples to explore the relationship between industrial robots and environmental pollution in China, providing references for other emerging countries to improve environmental quality using industrial robots. (2) In terms of theory, this paper is not limited to revealing the superficial relationship between industrial robots and environmental pollution. it starts from a new perspective and provides an in-depth analysis of how industrial robots affect environmental pollution through employment skill structure and green technology innovation. This not only enriches research in the fields of industrial robots and the environment, but is also of great significance in guiding the direction of industrial policy and technology research and development.

## Theoretical analysis and hypothesis

### Industrial robots and environmental pollution

**Figure 2:**
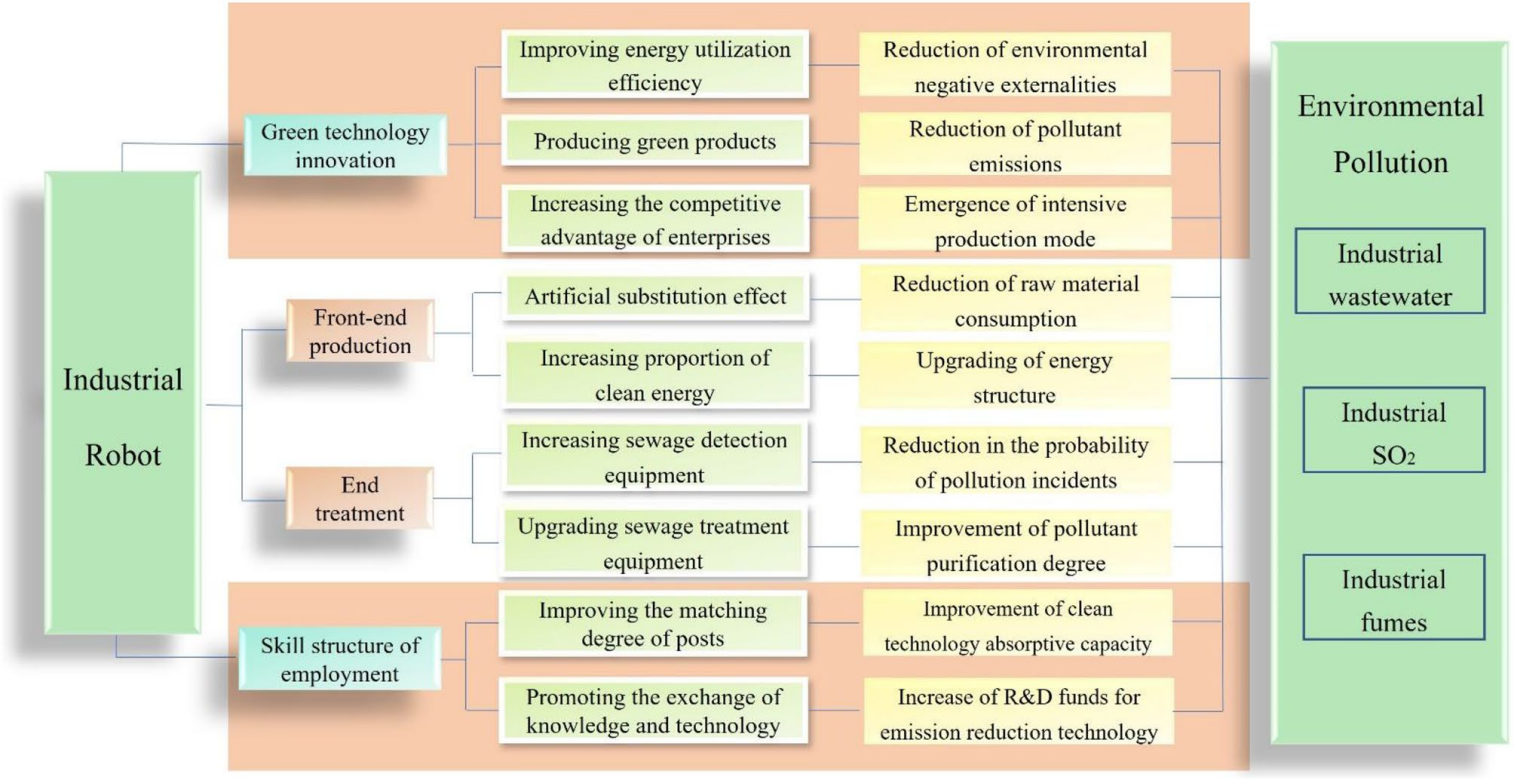
The impact of industrial robots on environmental pollution.

As shown in Fig. 2, the impact of industrial robots on environmental pollution is mainly reflected in two aspects: Front-end production and end treatment. In front-end production, industrial robots enable artificial substitution effects^25^. Manual operation is replaced by machine operation, reducing the raw materials needed for manual operation. Through the specific program setting of industrial robots, clean energy is applied to industrial production^26^. The use of traditional fuels such as coal and oil is reduced. In terms of end treatment, the traditional pollutant concentration tester only measures a single type of pollutant. Its data cannot be obtained in time. It is easy to cause pollution incidents. Industrial robots can measure a variety of pollutants, and have the function of remote unmanned operation and warning. It reflects the pollution situation in time, reducing the probability of pollution incidents. The use of robots can upgrade sewage treatment equipment and improve the accuracy of pollution treatment, reducing pollutant emissions. Based on the above analysis, this paper proposes hypothesis 1.

#### Hypothesis 1

The use of industrial robots can reduce environmental pollution.

### Mediating effect of green technology innovation

Industrial robots can affect environmental pollution by promoting green technology innovation. The transmission path of “industrial robots-green technology innovation-environmental pollution” is formed. Industrial robots are the materialization of technological progress in the field of enterprise R&D. Its impact on green technology innovation is mainly manifested in the following two aspects: Firstly, industrial robots classify known knowledge, which helps enterprises to integrate internal and external knowledge^27^. The development of green technology innovation activities of enterprises is promoted. Secondly, enterprises can simulate existing green technologies through industrial robots. The shortcomings of green technology in each link are found. Based on this, enterprises can improve and perfect green technology in a targeted manner. Industrial robots can collect and organize data, which enables enterprises to predict production costs and raw material consumption. Excessive procurement by enterprises can occupy working capital. Inventory backlog leads to warehousing, logistics and other expenses, increasing storage costs^28^. Forecasting the consumption of raw materials allows enterprises to purchase precisely, preventing over-procurement and inventory backlog, thereby reducing the use of working capital and storage costs^29^. The production cost of enterprises is reduced. Enterprises have more funds for green technology research and development.

The continuous innovation of green technology is helpful to solve the problem of environmental pollution. Firstly, green technology innovation helps use resources better^30^, lowers dependence on old energy, and reduces environmental damage. Secondly, green technology innovation promotes the greening of enterprises in manufacturing, sales and after-sales^31^. The emission of pollutants in production process is reduced. Finally, green technology innovation improves the advantages of enterprises in market competition^32^. The production possibility curve expands outward, which encourages enterprises to carry out intensive production. Based on the above analysis, this paper proposes hypothesis 2.

#### Hypothesis 2

Industrial robots can reduce environmental pollution through green technology innovation.

### Mediating effect of employment skill structure

Industrial robots can affect environmental pollution through employment skill structure. The transmission path of “industrial robots-employment skill structure-environmental pollution” is formed. Industrial robots have substitution effect and creation effect on the labor force, improving the employment skill structure. Regarding the substitution effect, enterprises use industrial robots to complete simple and repetitive tasks to improve production efficiency, which crowds out low-skilled labor^6^. Regarding the creation effect, industrial robots create a demand for new job roles that matches automation, such as robot engineers, data analysts, machine repairers, which increases the number of highly skilled labor^33^. The reduction of low-skilled labor and increase of high-skilled labor improve employment skill structure.

High-skilled labor is reflected in the level of education^34^. Its essence is to have a higher level of skills and environmental awareness, which is the key to reducing environmental pollution. Compared with low-skilled labor, high-skilled labor has stronger ability to acquire knowledge and understand skills, which improves the efficiency of cleaning equipment and promotes emission reduction. The interaction and communication between highly skilled labor is also crucial for emission reduction. The excessive wage gap between employees brings high communication costs, which hinders the exchange of knowledge and technology between different employees. The increase in the proportion of high-skilled labor can solve this problem and improve the production efficiency of enterprises^35^. The improvement of production efficiency enables more investment in emission reduction research, decreasing pollutant emissions. Based on the above analysis, this paper proposes hypothesis 3.

#### Hypothesis 3

Industrial robots can reduce environmental pollution by optimizing employment skills structure.

## Model construction and variable selection

### Model construction

#### Panel data model

The panel data model is a significant statistical method, first introduced by Mundlak^36^. Subsequently, numerous scholars have used this model to examine the baseline relationships between core explanatory variables and explained variables^37^. To test the impact of industrial robots on environmental pollution, this paper sets the following panel data model:

1 $$\ln Yit = \alpha 0 + \alpha 1 \times \ln IRit + \alpha 2 \times \ln Xit + \lambda i + \varphi t + \varepsilon it$$

In formula (1), *Y*_*it*_ is the explained variable, indicating the degree of environmental pollution in region *i* in year *t*. *IR*_*it*_ is the core explanatory variable, indicating the installation density of industrial robots in region *i* in year *t*.* X*_*it*_ is a series of control variables, including economic development level (GDP), urbanization level (URB), industrial structure (EC), government intervention (GOV) and environmental regulation (ER). $\lambda i$ is the regional factor. $\varphi t$ is the time factor. $\varepsilon it$ is the disturbance term.

#### Mediating effect model

To test the transmission mechanism of industrial robots affecting environmental pollution, this paper sets the following mediating effect model:

2 $$\ln Mit = \beta 0 + \beta 1 \times \ln IRit + \beta 2 \times \ln Xit + \lambda i + \varphi t + \varepsilon it$$

3 $$\ln Yit = \theta 0 + \theta 1 \times \ln IRit + \theta 2 \times \ln Mit + \theta 3 \times \ln Xit + \lambda i + \varphi t + \varepsilon it$$

In formula (2), *M* is the mediating variable, which mainly includes green technology innovation and employment skill structure. Formula (2) measures the impact of industrial robots on mediating variables. Formula (3) measures the impact of intermediary variables on environmental pollution. According to the principle of mediating effect^38^, the direct effect $\theta 1$, mediating effect $\beta 1 \times \theta 2$ and total effect $\alpha 1$ satisfy $\alpha 1 = \theta 1 + \beta 1 \times \theta 2$.

#### Panel quantile model

The panel quantile model was first proposed by Koenke and Bassett^39^. It is mainly used to analyze the impact of core explanatory variables on the explained variables under different quantiles^40^. To empirically test the heterogeneous impact of industrial robots on environmental pollution under different levels of environmental pollution, this paper sets the following panel quantile model:

4 $$Q\tau (\ln Yit) = \gamma 0 + \gamma 1 \times \ln IRit + \gamma 2 \times \ln Xit + \lambda i + \varphi t + \varepsilon it$$

In formula (4), $\tau$ represents the quantile value. $\gamma 1$ reflects the difference in the impact of industrial robots on environmental pollution at different quantiles. $\gamma 2$ indicates the different effects of control variables at different quantiles.

### Variable selection

#### Explained variable

The explained variable is environmental pollution. Considering the timeliness and availability of data, this paper selects industrial wastewater discharge, industrial SO_2_ emissions and industrial soot emissions as indicators of environmental pollution.

#### Explanatory variable

According to production theory, industrial robots can enhance production efficiency^41^. Efficient production implies reduced energy wastage, which in turn decreases the emission of pollutants. Industrial robots can upgrade pollution control equipment, heightening the precision in pollution treatment and reducing pollutant discharge. Referring to Acemoglu and Restrepo^25^, this paper selects the installation density of industrial robots as a measure. The specific formula is as follows:

5 $$IRit = \sum\limits_{j} {\frac{Laborji}{{\sum\limits_{j} {Laborji} }}} \times IRjt$$

In formula (5), *Labor*_*ji*_ is the number of labor force in industry *j* in region *i*. *IR*_*jt*_ is the stock of industrial robot use in industry *j* in the year *t*.

#### Mediating variable

1. Green technology innovation. Industrial robots can increase the demand for highly-skilled labor^42^, subsequently influencing green technology innovation. Compared to ordinary labor, highly-skilled labor possesses a richer knowledge base and technological learning capability, improving the level of green technology innovation. Green technology innovation can improve energy efficiency^43^, reducing pollution generated by energy consumption. The measurement methods of green technology innovation mainly include three kinds: The first method is to use simple technology invention patents as measurement indicators. Some of technical invention patents are not applied to the production process of enterprise, they cannot fully reflect the level of technological innovation. The second method is to use green product innovation and green process innovation as measurement indicators. The third method is to use the number of green patent applications or authorizations as a measure^44^. This paper selects the number of green patent applications as a measure of green technology innovation.
2. Employment skill structure. The use of industrial robots reduces the demand for labor performing simple repetitive tasks and increases the need for engineers, technicians, and other specialized skilled personnel, improving the employment skill structure^45^. Compared to ordinary workers, highly-skilled laborers typically have a stronger environmental awareness^46^. Such environmental consciousness may influence corporate decisions, prompting companies to adopt eco-friendly production methods, thus reducing environmental pollution. There are two main methods to measure the structure of employment skills: One is to use the proportion of employees with college degree or above in the total number of employees as a measure. The other is to use the proportion of researchers as a measure. The educational level can better reflect the skill differences of workers. This paper uses the first method to measure the employment skill structure.

#### Control variable

1. Economic development level. According to the EKC hypothesis^47^, in the initial stage of economic development, economic development mainly depends on input of production factors, which aggravates environmental pollution. With the continuous development of economy, people begin to put forward higher requirements for environmental quality. The government also begins to adopt more stringent policies to control environmental pollution, which can reduce the level of environmental pollution. According to Liu and Lin^48^, This paper uses per capita GDP to measure economic development level.
2. Urbanization level. The improvement of urbanization level has both positive and negative effects on pollution. Urbanization can improve the agglomeration effect of cities. The improvement of agglomeration effect can not only promote the sharing of public resources such as infrastructure, health care, but also facilitate the centralized treatment of pollution. The efficiency of environmental governance is improved^49^. The acceleration of urbanization can increase the demand for housing, home appliances and private cars, which increases pollutant emissions^50^. This paper uses the proportion of urban population to total population to measure the level of urbanization.
3. Industrial structure. Industrial structure is one of the key factors that determine the quality of a country’s environmental conditions^51^. The increase in the proportion of capital and technology-intensive industries can effectively improve resource utilization efficiency and improve resource waste^52^. This paper selects the ratio of the added value of the tertiary industry to the secondary industry to measure industrial structure.
4. Government intervention. Government intervention mainly affects environmental pollution from the following two aspects: Firstly, the government can give high-tech, energy-saving and consumption-reducing enterprises relevant preferential policies, which promotes the development of emission reduction technologies for these enterprises^53^. Secondly, the government strengthens environmental regulation by increasing investment in environmental law enforcement funds, thus forcing enterprises to save energy and reduce emissions^54^. This paper selects the proportion of government expenditure in GDP to measure government intervention.
5. Environmental regulation. The investment in environmental pollution control is conducive to the development of clean and environmental protection technology, optimizing the process flow and improving the green production efficiency of enterprises^55^. Pollutant emissions are reduced. This paper selects the proportion of investment in pollution control to GDP to measure environmental regulation.

#### Data sources and descriptive statistics

This paper selects the panel data of 30 provinces in China from 2006 to 2019 as the research sample. Among them, the installation data of industrial robots are derived from International Federation of Robotics (IFR). The data of labor force and employees with college degree or above are from *China Labor Statistics Yearbook*. Other data are from the *China Statistical Yearbook*. The descriptive statistics of variables are shown in Table 3. Considering the breadth of application and the reliability of analysis capabilities, this paper uses Stata 16 for regression analysis.

**Table 3:** Descriptive statistics.

	Variables	Definition	Obs	Mean	Std. dev	Min	Max
Explained variable	WW	Industrial wastewater discharge (taking logarithm)	420	10.6997	0.9707	8.5303	12.6828
	SO_2_	Industrial SO_2_ emissions (taking logarithm)	420	3.4732	1.1952	− 2.4304	5.1281
	SW	Industrial soot emissions (taking logarithm)	420	8.7745	1.0498	4.9904	10.8597
Explanatory variable	IR	Industrial robot installation density (taking logarithm)	420	7.3783	1.7594	2.6567	11.8745
Mediating variables	GI	Green technology innovation (taking logarithm)	420	7.5112	1.6160	2.6391	11.1163
	EM	Employment skill structure (taking logarithm)	420	2.5563	0.5966	1.1019	4.1304
Control variables	GDP	Economic development level (taking logarithm)	420	3.9786	0.2446	3.3127	4.4954
	URB	Urbanization level (taking logarithm)	420	3.6484	0.5217	0.1906	4.3143
	EC	Industrial structure (taking logarithm)	420	− 0.0324	0.4009	− 0.6949	1.6398
	GOV	Government intervention (taking logarithm)	420	3.0493	0.3960	2.1163	4.1406
	ER	Environmental regulation (taking logarithm)	420	0.2332	0.5160	− 1.6825	1.5315

## Results analysis

### Spatial and temporal characteristics of environmental pollution and industrial robots in China

#### Environmental pollution

**Figure 3:**
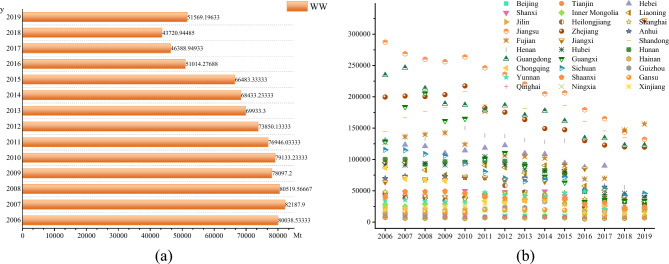
Industrial wastewater discharge from 2006 to 2019.

Figure 3a shows the overall trend of average industrial wastewater discharge in China from 2006 to 2019. From 2006 to 2019, the discharge of industrial wastewater shows a fluctuating downward trend, mainly due to the improvement of wastewater treatment facilities and the improvement of treatment capacity. Figure 3b shows the changing trend of average industrial wastewater discharge in 30 provinces of China from 2006 to 2019. Industrial wastewater discharge in most provinces has declined. There are also some provinces such as Fujian, Guizhou and Qinghai, which have increased industrial wastewater discharge. Their emission reduction task is very arduous.

**Figure 4:**
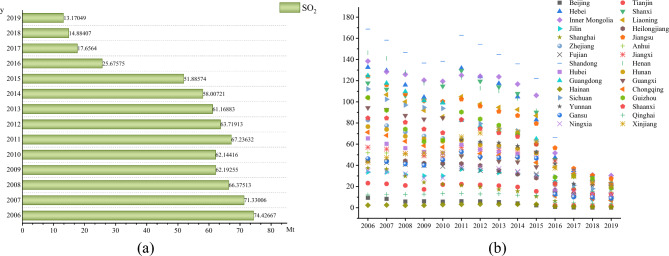
Industrial SO_2_ emissions from 2006 to 2019.

Figure 4a shows the overall trend of average industrial SO_2_ emissions in China from 2006 to 2019. From 2006 to 2019, industrial SO_2_ emissions shows a fluctuating downward trend, indicating that air pollution control and supervision are effective. Figure 4b shows the trend of average industrial SO_2_ emissions in 30 provinces of China from 2006 to 2019. Similar to industrial wastewater, industrial SO_2_ emissions decrease in most provinces.

**Figure 5:**
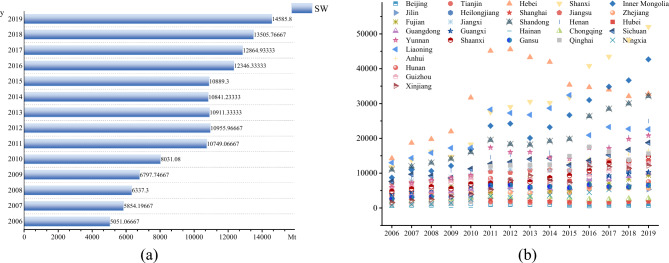
Industrial soot emissions from 2006 to 2019.

Figure 5a shows the overall trend of average industrial soot emissions in China from 2006 to 2019. Different from industrial wastewater and industrial SO_2_, the emission of industrial soot is increasing year by year. From the perspective of governance investment structure, compared with industrial wastewater and industrial SO_2_, the investment proportion of industrial soot is low. From the perspective of source, industrial soot mainly comes from urban operation, industrial manufacturing and so on. The acceleration of urbanization and the expansion of manufacturing scale have led to an increase in industrial soot emissions. Figure 5b shows the trend of industrial soot emissions in 30 provinces in China from 2006 to 2019. The industrial soot emissions in most provinces have increased.

**Figure 6:**
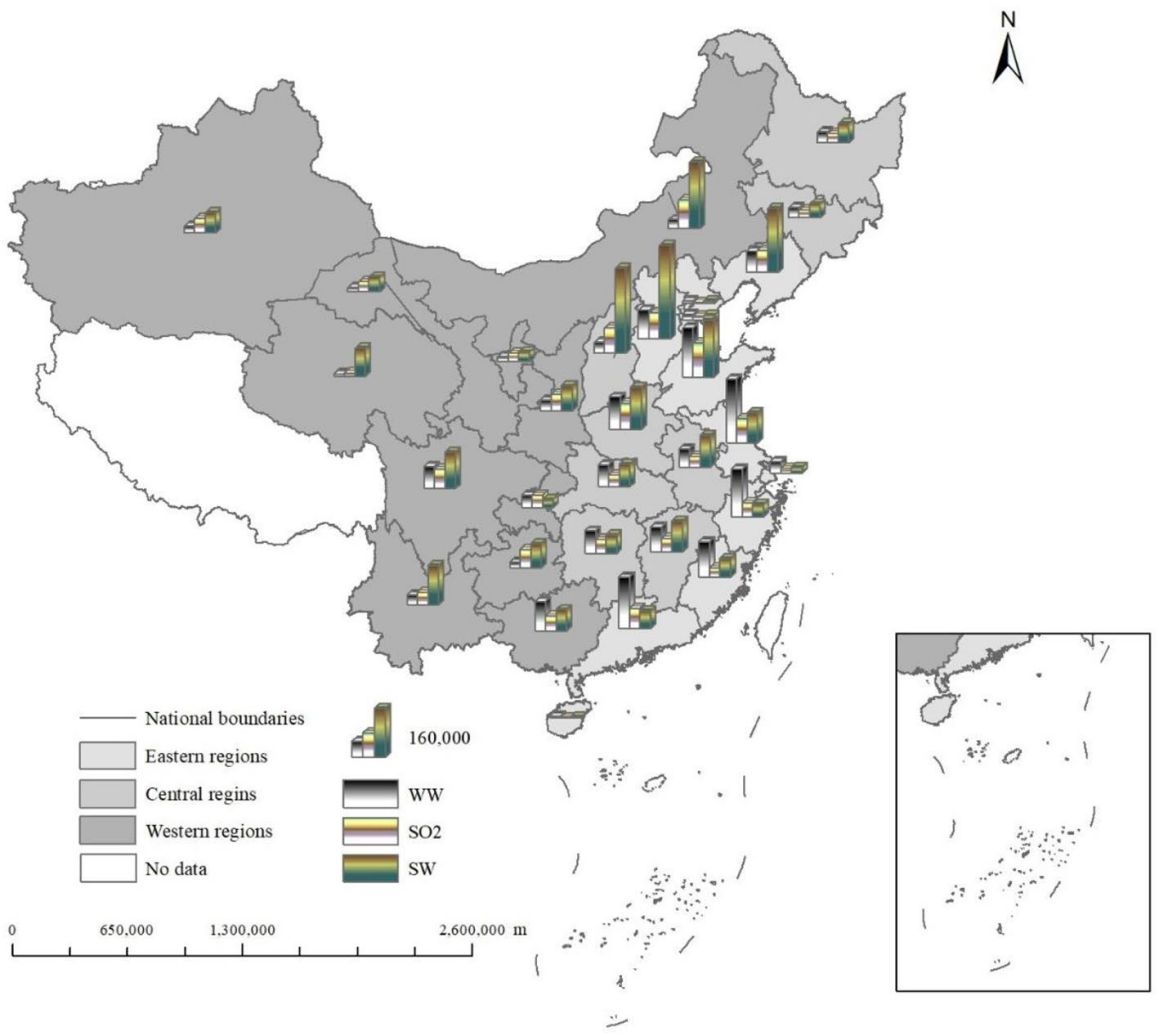
Spatial distribution characteristics of industrial wastewater, industrial SO_2_ and industrial soot.

Figure 6 shows the spatial distribution characteristics of industrial wastewater, industrial SO_2_ and industrial soot emissions. The three types of pollutant emissions in the central region are the largest, followed by the eastern region, and the three types of pollutant emissions in the western region are the smallest. Due to resource conditions and geographical location, the central region is mainly dominated by heavy industry. The extensive development model of high input and consumption makes its pollutant emissions higher than the eastern and western regions. The eastern region is mainly capital-intensive and technology-intensive industries, which makes its pollutant emissions lower than the central region. Although the leading industry in the western region is heavy industry, its factory production and transportation scale are not large, which produces less pollutants.

#### Industrial robots

**Figure 7:**
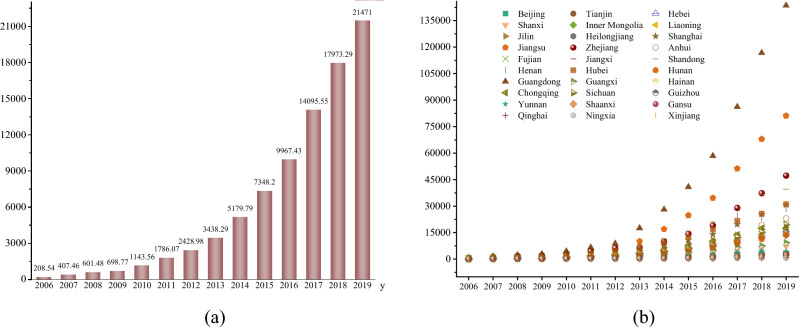
Installation density of industrial robots from 2006 to 2019.

Figure 7a shows the overall trend of installation density of industrial robots in China from 2006 to 2019. From 2006 to 2019, the installation density of industrial robots in China shows an increasing trend year by year. The increase of labor cost and the decrease of industrial robot cost make enterprises use more industrial robots, which has a substitution effect on labor force. The installation density of industrial robots is increased. Figure 7b shows the trend of installation density of industrial robots in 30 provinces of China from 2006 to 2019. The installation density of industrial robots in most provinces has increased. Among them, the installation density of industrial robots in Guangdong Province has the largest growth rate. The installation density of industrial robots in Heilongjiang Province has the smallest growth rate.

**Figure 8:**
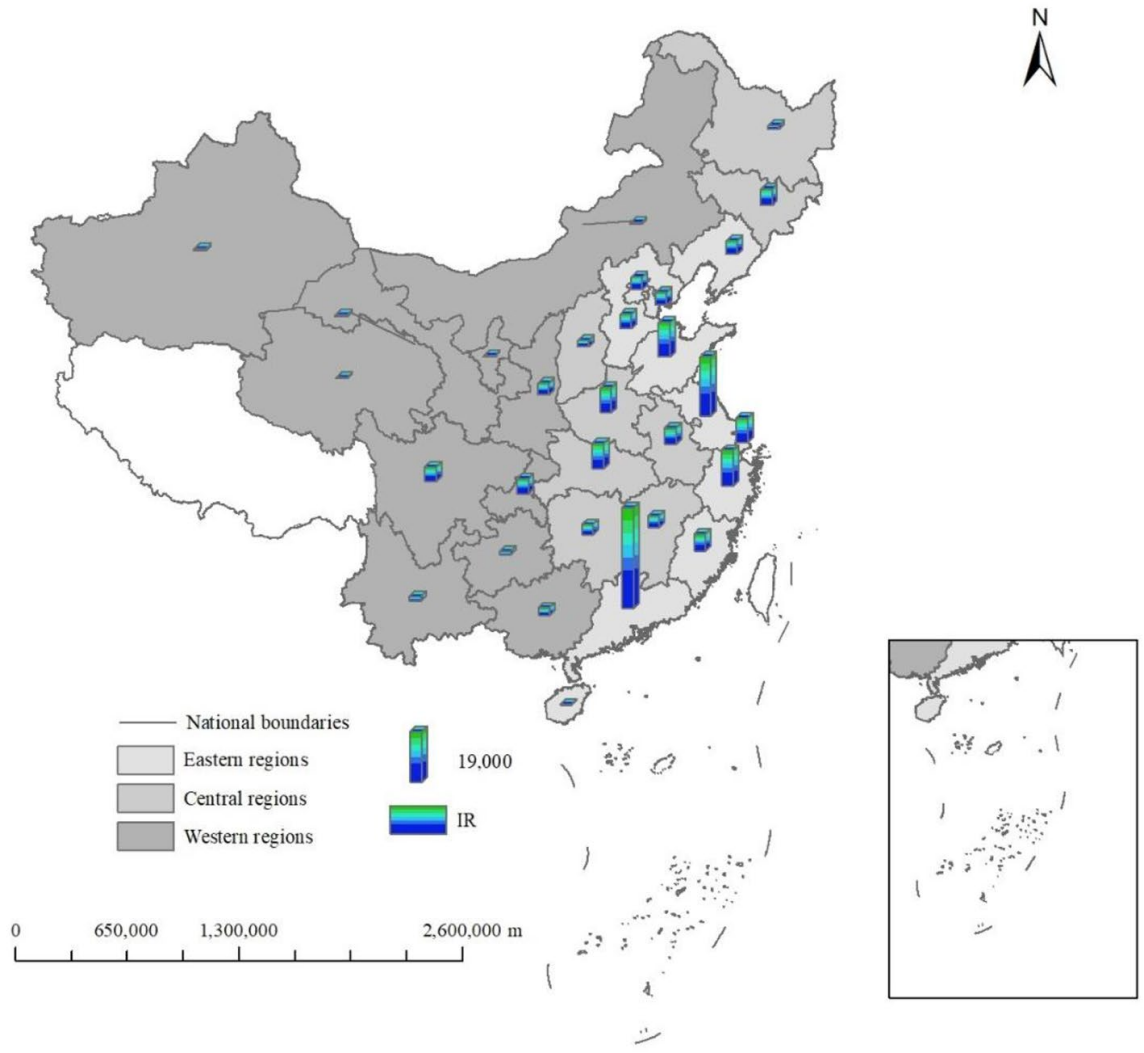
Spatial distribution characteristics of industrial robots.

Figure 8 shows the spatial distribution characteristics of installation density of industrial robots. The installation density of industrial robots in the eastern region is the largest, followed by the central region, and the installation density of industrial robots in the western region is the smallest. The eastern region is economically developed and attracts lots of talents to gather here, which provides talent support for the development of industrial robots. Advanced technology also leads to the rapid development of industrial robots in the eastern region. The economy of western region is backward, which inhibits the development of industrial robots.

### Benchmark regression results

Table 4 reports the estimation results of the ordinary panel model. Among them, the F test and LM test show that the mixed OLS model should not be used. The Hausman test shows that the fixed effect model should be selected in the fixed effect model and random effect model. This paper selects the estimation results of the fixed effect model to explain.

**Table 4:** Benchmark regression results.

	lnWW			lnSO_2_			lnSW		
	OLS	FE	RE	OLS	FE	RE	OLS	FE	RE
lnIR	0.227***	− 0.242***	0.0707***	0.0566**	− 0.0875*	− 0.175***	0.458***	− 0.277***	0.0732***
	(14.15)	(− 4.64)	(3.07)	(2.30)	(− 1.77)	(− 5.06)	(18.38)	(− 4.98)	(2.79)
lnGDP	− 0.0395	− 0.0299	− 0.0542	− 0.188**	− 0.0984*	− 0.157**	0.0105	− 0.0464	− 0.148***
	(− 0.81)	(− 0.65)	(− 1.06)	(− 2.53)	(− 1.76)	(− 1.99)	(0.14)	(− 0.95)	(− 2.80)
lnURB	− 0.927***	− 0.814***	− 0.698***	− 1.146***	2.994***	− 0.0311	− 1.645***	1.443***	1.435***
	(− 5.60)	(− 2.92)	(− 3.11)	(− 4.51)	(8.80)	(− 0.09)	(− 6.39)	(4.86)	(5.84)
lnEC	− 0.749***	− 0.105	− 0.444***	− 1.581***	0.160	− 1.557***	− 1.108***	− 0.217*	− 0.302***
	(− 10.07)	(− 0.96)	(− 5.10)	(− 13.85)	(1.19)	(− 11.51)	(− 9.59)	(− 1.86)	(− 3.38)
lnGOV	− 1.564***	− 0.435***	− 0.813***	− 1.081***	0.202	− 0.153	0.190	0.0530	0.605***
	(− 20.58)	(− 2.70)	(− 6.76)	(− 9.27)	(1.02)	(− 0.85)	(1.61)	(1.08)	(4.39)
lnER	− 0.0295	0.110***	0.155***	0.347***	0.0066	0.253***	0.567***	0.0481	0.0866*
	(− 0.61)	(2.82)	(3.41)	(4.67)	(0.14)	(3.56)	(7.54)	(1.16)	(1.92)
Constant	17.85***	16.62***	15.91***	12.61***	− 6.509***	6.777***	11.09***	3.011**	2.094*
	(28.38)	(11.95)	(16.16)	(13.06)	(− 3.83)	(4.56)	(11.35)	(2.03)	(1.90)
F test		35.47***			52.12***			94.77***	
Hausman test		167.14***			132.54***			187.70***	
LM test			566.30***			668.45***			1124.72***
R^2^	0.7883	0.5124	0.3138	0.6712	0.8694	0.6737	0.5633	0.6610	0.5982
N	420	420	420	420	420	420	420	420	420

*t* statistics in parentheses.

***p < 0.01, **p < 0.05, *p < 0.1.

Regarding the core explanatory variable, industrial robots have a significant negative impact on the emissions of industrial wastewater, industrial SO_2_ and industrial soot. Specifically, industrial robots have the greatest negative impact on industrial soot emissions, with a coefficient of -0.277 and passing the 1% significance level. The negative impact of industrial robots on industrial wastewater discharge is second, with an estimated coefficient of -0.242, which also passes the 1% significance level. The negative impact of industrial robots on industrial SO_2_ emissions is the smallest, with an estimated coefficient of -0.0875 and passing the 10% significant level. Compared with industrial wastewater and SO_2_, industrial robots have some unique advantages in reducing industrial soot emissions. Firstly, in terms of emission sources, industrial soot emissions mainly come from physical processes such as cutting. These processes can be significantly improved through precise control of industrial robots. Industrial SO_2_ comes from the combustion process. Industrial wastewater originates from various industrial processes. It is difficult for industrial robots to directly control these processes. Secondly, in terms of source control and terminal treatment, industrial robots can reduce excessive processing and waste of raw materials, thereby controlling industrial soot emissions at the source. For industrial SO_2_ and industrial wastewater, industrial robots mainly play a role in terminal treatment. Since the terminal treatment of industrial SO_2_ and industrial wastewater often involves complex chemical treatment processes, it is difficult for industrial robot technology to fully participate in these processes. This makes the impact of industrial robots in the field of industrial SO_2_ and industrial wastewater more limited than that in the field of industrial soot.

Regarding the control variables, the level of economic development has a significant inhibitory effect on industrial SO_2_ emissions. The higher the level of economic development, the stronger the residents’ awareness of environmental protection, which constrains the pollution behavior of enterprises. The government also adopts strict policies to control pollutant emissions. The impact of urbanization level on the discharge of industrial wastewater, industrial SO_2_ and industrial soot is significantly negative. The improvement of urbanization level can improve the efficiency of resource sharing and the centralized treatment of pollutants, reducing environmental pollution. The industrial structure significantly reduces industrial SO_2_ and industrial soot emissions. The upgrading of industrial structure not only reduces the demand for energy, but also improves the efficiency of resource utilization. The degree of government intervention only significantly reduces the discharge of industrial wastewater. The possible reason is that to promote economic development, the government invests more money in high-yield areas, which crowds out investment in the environmental field. Similar to the degree of government intervention, environmental regulation has a negative impact on industrial wastewater discharge. The government’s environmental governance investment has not given some support to the enterprise’s clean technology research, which makes the pollution control investment not produce good emission reduction effect.

### Mediation effect regression results

#### Green technology innovation

Table 5 reports the results of intermediary effect model when green technology innovation is used as an intermediary variable. Industrial robots can have a positive impact on green technology innovation. For every 1% increase in the installation density of industrial robots, the level of green technology innovation increases by 0.722%. After adding the green technology innovation, the estimated coefficient of industrial robots has decreased, which shows that the intermediary variable is effective.

**Table 5:** Regression results of mediating effect model (green technology innovation).

	lnGI	lnWW	lnSO_2_	lnSW	lnWW	lnSO_2_	lnSW
lnIR	0.722***	− 0.222**	− 0.069*	− 0.267***	− 0.242***	− 0.0794*	− 0.277***
	(6.67)	(− 4.02)	(− 1.76)	(− 4.13)	(− 4.64)	(− 1.77)	(− 4.98)
GI		− 0.0274*	− 0.0143*	− 0.0143*			
		(1.82)	(− 1.88)	(1.77)			
lnGDP	− 2.231***	− 0.551*	1.628***	0.842***	− 0.0299	− 0.0984*	− 0.0464
	(− 3.58)	(− 1.84)	(5.36)	(2.75)	(− 0.65)	(− 1.76)	(− 0.95)
lnURB	− 0.609***	− 0.107	0.882***	0.431***	− 0.814***	2.994***	1.443***
	(− 4.38)	(− 1.59)	(12.90)	(6.26)	(− 2.92)	(8.80)	(4.86)
lnEC	− 0.316	− 0.068	0.0361	− 0.269**	− 0.105	0.160	− 0.217*
	(− 1.37)	(− 0.62)	(0.33)	(− 2.41)	(− 0.96)	(1.19)	(− 1.86)
lnGOV	0.0980	− 0.370**	− 0.0072	0.428***	− 0.435***	0.202	0.530***
	(0.29)	(− 2.36)	(− 0.05)	(2.67)	(− 2.70)	(1.02)	(3.08)
lnER	− 0.0731	0.102***	0.0999**	0.0939**	0.110***	0.0066	0.0481
	(− 0.88)	(2.60)	(2.51)	(2.35)	(2.82)	(0.14)	(1.16)
Constant	6.996***	15.71***	− 5.180***	3.497***	16.62***	− 6.509***	3.011**
	(2.60)	(12.22)	(− 3.98)	(2.67)	(11.95)	(− 3.83)	(2.03)
R^2^	0.5755	0.5181	0.9112	0.6933	0.5124	0.8694	0.6610
N	420	420	420	420	420	420	420

*t* statistics in parentheses.

***p < 0.01, ** *p* < 0.05, * *p* < 0.1.

In the impact of industrial robots on industrial wastewater discharge, the mediating effect of green technology innovation accounts for 8.17% of the total effect. In the impact of industrial robots on industrial SO_2_ emissions, the mediating effect of green technology innovation accounts for 11.8% of the total effect. In the impact of industrial robots on industrial soot emissions, the mediating effect of green technology innovation accounts for 3.72% of the total effect.

#### Employment skill structure

Table 6 reports the results of intermediary effect model when the employment skill structure is used as an intermediary variable. Industrial robots have a positive impact on the employment skill structure. For every 1% increase in the installation density of industrial robots, the employment skill structure is improved by 0.0837%. Similar to green technology innovation, the intermediary variable of employment skill structure is also effective.

**Table 6:** Regression results of mediating effect model (employment skill structure).

	lnEM	lnWW	lnSO_2_	lnSW	lnWW	lnSO_2_	lnSW
lnIR	0.0837**	− 0.236***	− 0.0694*	− 0.234***	− 0.252***	− 0.0875*	− 0.277***
	(2.58)	(− 4.59)	(− 1.71)	(− 4.19)	(− 4.64)	(− 1.77)	(− 4.98)
lnEM		− 0.201**	− 0.216**	− 0.514***			
		(− 2.46)	(2.01)	(− 3.04)			
lnGDP	1.563***	− 0.299	1.658***	0.804**	− 0.0299	− 0.0984*	− 0.0464
	(8.39)	(− 0.94)	(5.09)	(2.45)	(− 0.65)	(− 1.76)	(− 0.95)
lnURB	0.0209	− 0.120*	0.891***	0.423***	− 0.814***	2.994***	1.443***
	(0.50)	(− 1.83)	(13.34)	(6.29)	(− 2.92)	(8.80)	(4.86)
lnEC	0.0863	− 0.0593	0.0405	− 0.274**	− 0.105	0.160	− 0.217*
	(1.25)	(− 0.55)	(0.37)	(− 2.46)	(− 0.96)	(1.19)	(− 1.86)
lnGOV	0.326***	− 0.302*	− 0.009	0.429***	− 0.435***	0.202	0.530***
	(3.28)	(− 1.91)	(− 0.06)	(2.63)	(− 2.70)	(1.02)	(3.08)
lnER	− 0.0823***	0.0833**	0.101**	0.0931**	0.110***	0.0066	0.0481
	(− 3.33)	(2.11)	(2.51)	(2.29)	(2.82)	(0.14)	(1.16)
Constant	− 5.372***	14.82***	− 5.274***	3.616***	16.62***	− 6.509***	3.011**
	(− 6.67)	(11.06)	(− 3.86)	(2.63)	(11.95)	(− 3.83)	(2.03)
R^2^	0.8991	0.5243	0.9111	0.6930	0.5124	0.8694	0.6610
N	420	420	420	420	420	420	420

*t* statistics in parentheses.

***p < 0.01, **p < 0.05, *p < 0.1.

In the impact of industrial robots on industrial wastewater discharge, the mediating effect of employment skill structure accounts for 6.67% of the total effect. In the impact of industrial robots on industrial SO_2_ emissions, the mediating effect of employment skill structure accounts for 20.66% of the total effect. In the impact of industrial robots on industrial soot emissions, the mediating effect of employment skill structure accounts for 15.53% of the total effect.

### Robustness test and endogeneity problem

#### Robustness test

To ensure the robustness of the regression results, this paper tests the robustness by replacing core explanatory variables, shrinking tail and replacing sample. Regarding the replacement of core explanatory variables, in the benchmark regression, the installation density of industrial robots is measured by the stock of industrial robots. Replacing the industrial robot stock with the industrial robot installation quantity, this paper re-measures the industrial robot installation density. Regarding the tail reduction processing, this paper reduces the extreme outliers of all variables in the upper and lower 1% to eliminate the influence of extreme outliers. Regarding the replacement of samples, this paper removes the four municipalities from the sample. The estimation results are shown in Table 7. Industrial robots still have a significant negative impact on environmental pollution, which confirms the robustness of benchmark regression results.

**Table 7:** Robustness test.

	Replacing core explanatory variables			Shrinking tail			Replacing sample		
	lnWW	lnSO_2_	lnSW	lnWW	lnSO_2_	lnSW	lnWW	lnSO_2_	lnSW
lnIR	− 0.221***	− 0.0456*	− 0.256***	− 0.277***	− 0.128**	− 0.288***	− 0.152***	− 0.206***	− 0.303***
	(− 4.51)	(− 1.92)	(− 5.15)	(− 5.69)	(− 2.57)	(− 5.68)	(− 2.66)	(− 4.16)	(− 5.24)
lnGDP	− 0.518*	1.717***	0.893**	− 0.874***	1.629***	1.389***	− 0.0310	− 0.108***	− 0.0485
	(− 1.77)	(5.80)	(3.00)	(− 3.16)	(5.75)	(4.83)	(− 0.66)	(− 2.67)	(− 1.02)
lnURB	− 0.106	0.896***	0.442***	− 0.127	0.678***	0.497***	− 0.0331	− 0.251	− 0.503
	(− 1.61)	(13.42)	(6.58)	(− 1.34)	(6.95)	(5.02)	(− 0.09)	(− 0.78)	(− 1.34)
lnEC	− 0.0503	0.0633	− 0.255**	− 0.0966	0.0056	− 0.167	− 0.0429	− 0.132	− 0.288**
	(− 0.46)	(0.58)	(− 2.31)	(− 0.94)	(0.05)	(− 1.56)	(− 0.36)	(− 1.27)	(− 2.38)
lnGOV	− 0.374**	− 0.0008	0.417***	− 0.474***	− 0.0308	0.287*	− 0.466**	− 0.263	0.247
	(− 2.36)	(− 0.01)	(2.59)	(− 3.10)	(− 0.20)	(1.80)	(− 2.51)	(− 1.64)	(1.32)
lnER	0.106***	0.104***	0.0993**	0.112***	0.0898**	0.0601	0.112**	0.0264	0.106**
	(2.70)	(2.61)	(2.49)	(2.94)	(2.29)	(1.51)	(2.57)	(0.70)	(2.39)
Constant	15.34***	− 5.700***	3.161**	17.34***	− 4.052***	1.617	13.37***	7.818***	11.39***
	(12.28)	(− 4.52)	(2.49)	(14.12)	(− 3.22)	(1.27)	(7.63)	(5.16)	(6.45)
R^2^	0.5116	0.9108	0.6923	0.5364	0.9039	0.6926	0.5226	0.9063	0.7235
N	364	364	364	364	364	364	364	364	364

*t* statistics in parentheses.

***p < 0.01, **p < 0.05, *p < 0.1.

#### Endogeneity problem

Logically speaking, although the use of industrial robots can reduce environmental pollution, there may be reverse causality. Enterprises may increase the use of industrial robots to meet emission reduction standards, which increases the use of industrial robots in a region. Due to the existence of reverse causality, there is an endogenous problem that cannot be ignored between industrial robots and environmental pollution.

To solve the impact of endogenous problems on the estimation results, this paper uses the instrumental variable method to estimate. According to the selection criteria of instrumental variables, this paper selects the installation density of industrial robots in the United States as the instrumental variable. The trend of the installation density of industrial robots in the United States during the sample period is similar to that of China, which is consistent with the correlation characteristics of instrumental variables. The application of industrial robots in the United States is rarely affected by China’s economic and social factors, and cannot affect China’s environmental pollution, which is in line with the exogenous characteristics of instrumental variables.

Table 8 reports the estimation results of instrumental variable method. Among them, the column (1) is listed as the first stage regression result. The estimated coefficient of instrumental variable is significantly positive, which is consistent with the correlation. Column (2), column (3) and column (4) of Table 8 are the second stage regression results of industrial wastewater, industrial SO_2_ and industrial soot emissions as explanatory variables. The estimated coefficients of industrial robots are significantly negative, which again verifies the hypothesis that industrial robots can reduce environmental pollution. Compared with Table 4, the absolute value of estimated coefficient of industrial robots is reduced, which indicates that the endogenous problems caused by industrial robots overestimate the emission reduction effect of industrial robots. The test results prove the validity of the instrumental variables.

**Table 8:** Regression results of instrumental variable method.

		lnWW	lnSO_2_	lnSW
	(1)	(2)	(3)	(4)
lnIR		− 0.0742**	− 0.065*	− 0.118***
		(− 2.28)	(− 1.66)	(− 3.33)
lnGDP	− 1.3663***	− 0.369	0.228	1.345***
	(− 4.86)	(− 1.41)	(1.28)	(5.36)
lnURB	− 0.0516	− 0.115***	0.475**	0.443***
	(− 0.60)	(− 2.69)	(2.44)	(5.78)
lnEC	− 0.6326***	0.0361	− 0.159*	− 0.0259
	(− 5.43)	(0.38)	(− 1.65)	(− 0.28)
lnGOV	− 0.3277**	− 0.309*	− 0.112	0.558**
	(− 1.99)	(− 1.90)	(− 0.65)	(2.39)
lnER	− 0.0528	0.109**	0.0842*	0.113***
	(− 1.31)	(2.57)	(1.78)	(2.76)
lnIV	5.3579***			
	(28.38)			
R^2^	0.9735	0.5007	0.9022	0.6464
N	420	420	420	420
F test	670.59***			
Kleibergen-Paap rk LM statistic		71.253***	71.253***	71.253***
Cragg-Donald Wald F statistic		805.506	805.506	805.506
Stock-Yogo bias critical value		16.38(10%)	16.38(10%)	16.38(10%)

*t* statistics in parentheses.

***p < 0.01, **p < 0.05, *p < 0.1.

### Panel quantile regression results

Traditional panel data models might obscure the differential impacts of industrial robots at specific pollution levels. To address this issue, this paper uses a panel quantile regression model to empirically analyze the effects of industrial robots across different environmental pollution levels.

Table 9 shows that industrial robots have a negative impact on industrial wastewater discharge. With the increase of the quantile of industrial wastewater discharge, the regression coefficient of industrial robots shows a W-shaped change. Specifically, when the industrial wastewater discharge is in the 0.1 quantile, the regression coefficient of industrial robot is − 0.229, and it passes the 1% significant level. When the industrial wastewater discharge is in the 0.25 quantile, the impact of industrial robots on industrial wastewater discharge is gradually enhanced. Its regression coefficient decreases from − 0.229 to − 0.256. When the industrial wastewater discharge is in the 0.5 quantile, the regression coefficient of industrial robot increases from − 0.256 to − 0.152. When the industrial wastewater discharge is at the 0.75 quantile, the regression coefficient of industrial robot decreases from − 0.152 to − 0.211. When the industrial wastewater discharge is in the 0.9 quantile, the regression coefficient of industrial robot increases from − 0.211 to − 0.188. For every 1% increase in the installation density of industrial robots, the discharge of industrial wastewater is reduced by 0.188%.

**Table 9:** Regression results of panel quantile model (lnWW).

	Q10	Q25	Q50	Q75	Q90
lnIR	− 0.229***	− 0.256***	− 0.152*	− 0.211***	− 0.188**
	(− 3.01)	(− 5.12)	(− 1.87)	(− 2.85)	(− 2.55)
lnGDP	− 0.327	− 0.424	− 0.559	− 0.744*	− 0.261
	(− 0.60)	(− 1.00)	(− 1.55)	(− 1.91)	(− 0.59)
lnURB	− 0.156*	− 0.221***	− 0.121**	− 0.0981**	− 0.103
	(− 1.91)	(− 3.01)	(− 2.00)	(− 2.32)	(− 1.55)
lnEC	− 0.0632	− 0.158	− 0.182*	− 0.134	− 0.0254
	(− 0.53)	(− 1.31)	(− 1.75)	(− 0.91)	(− 0.18)
lnGOV	− 0.362*	− 0.234	− 0.135	− 0.250	− 0.0926
	(− 1.71)	(− 0.86)	(− 0.66)	(− 1.24)	(− 0.52)
lnER	0.0443	0.0322	0.0632	0.100**	0.0960
	(0.68)	(0.59)	(1.03)	(2.00)	(1.63)
Constant	13.06***	13.69***	13.33***	14.61***	11.93***
	(4.78)	(6.05)	(6.66)	(6.62)	(5.64)
R^2^	0.8504	0.8338	0.8255	0.8190	0.8065
N	420	420	420	420	420

*t* statistics in parentheses.

***p < 0.01, **p < 0.05, *p < 0.1.

When industrial wastewater discharge is at a low percentile, the use of industrial robots can replace traditional production methods, reducing energy waste and wastewater discharge. As industrial wastewater discharge increases, the production process becomes more complex. Industrial robots may be involved in high-pollution, high-emission productions, diminishing the robots’ emission-reducing effects. When industrial wastewater discharge reaches high levels, pressured enterprises seek environmentally friendly production methods and use eco-friendly industrial robots to reduce wastewater discharge. As wastewater discharge continues to rise, enterprises tend to prioritize production efficiency over emission control, weakening the negative impact of industrial robots on wastewater discharge. When wastewater discharge is at a high percentile, enterprises should balance production efficiency and environmental protection needs, by introducing eco-friendly industrial robots to reduce wastewater discharge.

Table 10 shows that with the increase of industrial SO_2_ emission quantile level, the negative impact of industrial robots on industrial SO_2_ emissions gradually increases. Specifically, when industrial SO_2_ emissions are below 0.5 quantile, the impact of industrial robots on industrial SO_2_ emissions is not significant. When the industrial SO_2_ emissions are above 0.5 quantile, the negative impact of industrial robots on industrial SO_2_ emissions gradually appears.

**Table 10:** Regression results of panel quantile model (lnSO_2_).

	Q10	Q25	Q50	Q75	Q90
lnIR	− 0.112	− 0.109	− 0.107	− 0.122*	− 0.139*
	(− 1.35)	(− 1.38)	(− 1.54)	(− 1.68)	(− 1.76)
lnGDP	1.001	1.059*	1.197***	1.131**	0.617
	(1.33)	(1.67)	(2.65)	(2.51)	(1.42)
lnURB	0.906***	0.903***	0.755***	0.357**	0.253**
	(7.46)	(6.88)	(4.50)	(2.01)	(2.17)
lnEC	− 0.0354	− 0.0599	− 0.0174	− 0.0160	− 0.0898
	(− 0.28)	(− 0.48)	(− 0.17)	(− 0.12)	(− 0.67)
lnGOV	0.0512	0.192	0.175	− 0.163	− 0.0840
	(0.25)	(0.99)	(0.83)	(− 0.74)	(− 0.45)
lnER	0.0858	0.0622	0.0487*	0.0333	0.0439
	(1.47)	(1.26)	(1.70)	(0.78)	(0.93)
Constant	− 5.027	− 5.607**	− 5.674**	− 2.948	− 0.344
	(− 1.46)	(− 1.97)	(− 2.43)	(− 1.09)	(− 0.15)
R^2^	0.8914	0.8563	0.8249	0.8100	0.8158
N	420	420	420	420	420

*t* statistics in parentheses.

***p < 0.01, **p < 0.05, *p < 0.1.

When industrial SO_2_ emissions are at a low percentile, the application of industrial robots primarily aims to enhance production efficiency, not to reduce SO_2_ emissions. Enterprises should invest in the development of eco-friendly industrial robots, ensuring they are readily available for deployment when a reduction in industrial SO_2_ emissions is necessary. As industrial SO_2_ emissions continue to rise, both the government and the public pay increasing attention to the issue of SO_2_ emissions. To meet stringent environmental standards, enterprises begin to use industrial robots to optimize the production process, reduce reliance on sulfur fuels, and consequently decrease SO_2_ emissions. Enterprises should regularly evaluate the emission reduction effectiveness of industrial robots, using the assessment data to upgrade and modify the robots’ emission reduction technologies.

Table 11 shows that with the increase of industrial soot emissions quantile level, the negative impact of industrial robots on industrial soot emissions gradually weakens. Specifically, when industrial soot emissions are below 0.75 quantile, industrial robots have a significant negative impact on industrial soot emissions. This negative effect decreases with the increase of industrial soot emissions. When the industrial soot emissions are above 0.75 quantile, the negative impact of industrial robots on industrial soot emissions gradually disappears.

**Table 11:** Regression results of panel quantile model (lnSW).

	Q10	Q25	Q50	Q75	Q90
lnIR	− 0.261**	− 0.243***	− 0.129**	− 0.089**	− 0.104
	(− 2.32)	(− 2.99)	(− 2.52)	(− 2.01)	(− 1.46)
lnGDP	0.477	0.820	0.979**	0.749	0.298
	(1.16)	(1.62)	(2.10)	(1.64)	(0.57)
lnURB	0.610***	0.635***	0.448***	0.435***	0.485***
	(2.85)	(4.15)	(4.07)	(5.16)	(4.31)
lnEC	− 0.425***	− 0.339***	− 0.157	− 0.153*	− 0.143
	(− 2.87)	(− 3.14)	(− 1.43)	(− 1.78)	(− 1.20)
lnGOV	− 0.016	0.0977	0.101	0.188	0.0153
	(− 0.07)	(0.75)	(0.66)	(0.99)	(0.07)
lnER	0.0816*	0.124***	0.116**	0.0862	0.0327
	(1.70)	(3.84)	(2.24)	(1.29)	(0.59)
Constant	4.303**	2.444	1.584	2.296	4.710**
	(2.18)	(1.08)	(0.72)	(1.18)	(1.99)
R^2^	0.8796	0.8466	0.8227	0.8300	0.8498
N	420	420	420	420	420

*t* statistics in parentheses.

***p < 0.01, **p < 0.05, *p < 0.1.

When industrial soot emissions are at a low percentile, they come from a few sources easily managed by industrial robots. As industrial soot emissions increase, the sources become more diverse and complex, making it harder for industrial robots to control. Even with growing environmental awareness, it may take time to effectively use robots in high-emission production processes and control industrial soot emissions. Enterprises should focus on researching how to better integrate industrial robot technology with production processes that have high soot emission levels. The government should provide financial and technical support to enterprises, assisting them in using industrial robots more effectively for emission reduction.

**Figure 9:**
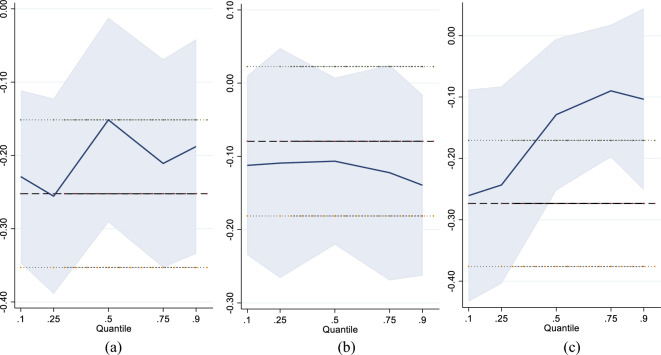
Change of quantile regression coefficient.

Figure 9 intuitively reflects the trend of the regression coefficient of industrial robots with the changes of industrial wastewater, industrial SO_2_ and industrial soot emissions. Figure 9a shows that with the increase of industrial wastewater discharge, the regression coefficient of industrial robots shows a W-shaped trend. Figure 9b shows that with the increase of industrial SO_2_ emissions, the regression coefficient of industrial robots gradually decreases. The negative impact of industrial robots on industrial SO_2_ emissions is gradually increasing. Figure 9c shows that with the increase of industrial soot emissions, the regression coefficient of industrial robots shows a gradual increasing trend. The negative impact of industrial robots on industrial soot emissions has gradually weakened. Figure 9a, b and c confirm the estimation results of Tables 9, 10 and 11.

### Heterogeneity analysis

#### Regional heterogeneity

This paper divides China into three regions: Eastern, central and western regions according to geographical location. The estimated results are shown in Table 12. The industrial robots in eastern region have the greatest negative impact on three pollutants, followed by central region, and the industrial robots in western region have the least negative impact on three pollutants. The use of industrial robots in eastern region far exceeds that in central and western regions. The eastern region is far more than central and western regions in terms of human capital, technological innovation and financial support. Compared with central and western regions, the artificial substitution effect, upgrading of sewage treatment equipment and improvement of energy utilization efficiency brought by industrial robots in eastern region are more obvious.

**Table 12:** Regression results based on regional heterogeneity.

	Eastern regions			Central regions			Western regions		
	lnWW	lnSO_2_	lnSW	lnWW	lnSO_2_	lnSW	lnWW	lnSO_2_	lnSW
lnIR	− 0.472***	− 0.441***	− 0.624***	− 0.275*	− 0.235*	− 0.128*	− 0.152**	− 0.182	− 0.116
	(− 6.00)	(− 6.10)	(− 6.42)	(− 1.83)	(− 1.90)	(− 1.72)	(− 1.98)	(− 1.24)	(− 1.18)
lnGDP	0.776***	− 0.543	0.535*	0.561	0.393	− 0.321	0.0487	− 0.0290	0.105*
	(3.11)	(− 1.14)	(1.66)	(0.63)	(0.54)	(− 0.73)	(1.05)	(− 0.68)	(1.82)
lnURB	− 1.141***	6.818***	2.992***	− 0.167	− 0.874	0.536	1.642**	− 0.652	− 4.028***
	(− 3.18)	(9.92)	(6.46)	(− 0.18)	(− 1.15)	(1.16)	(2.56)	(− 1.11)	(− 5.07)
lnEC	0.103	0.469	0.207	0.428	0.208	0.0166	0.582***	− 0.0563	− 0.543**
	(0.52)	(1.24)	(0.81)	(1.51)	(0.89)	(0.12)	(3.17)	(− 0.33)	(− 2.39)
lnGOV	− 0.408**	− 0.632*	0.160	0.0091	0.261	− 0.617*	− 0.216	0.234	1.228***
	(− 2.13)	(− 1.72)	(0.65)	(0.01)	(0.43)	(− 1.69)	(− 0.84)	(0.99)	(3.84)
lnER	0.0068	0.0403	− 0.0500	− 0.0577	− 0.199**	0.0145	0.274***	0.165***	0.144*
	(0.15)	(0.47)	(− 0.87)	(− 0.55)	(− 2.30)	(0.28)	(4.59)	(3.02)	(1.95)
Constant	9.738**	− 17.66***	− 10.57**	5.110	1.882	11.93**	6.683**	7.818***	20.36***
	(2.94)	(− 2.79)	(− 2.48)	(0.56)	(0.25)	(2.63)	(2.35)	(3.00)	(5.79)
R^2^	0.6131	0.9071	0.5698	0.6153	0.9157	0.8724	0.6994	0.9225	0.8130
N	154	154	154	112	112	112	154	154	154

*t* statistics in parentheses.

***p < 0.01, **p < 0.05, *p < 0.1.

#### Time heterogeneity

The development of industrial robots is closely related to policy support^56^. In 2013, the Ministry of Industry and Information Technology issued the “*Guiding Opinions on Promoting the Development of Industrial Robot Industry*”. This document proposes: By 2020, 3 to 5 internationally competitive leading enterprises and 8 to 10 supporting industrial clusters are cultivated. In terms of high-end robots, domestic robots account for about 45% of the market share, which provides policy support for the development of industrial robots. Based on this, this paper divides the total sample into two periods: 2006–2012 and 2013–2019, and analyzes the heterogeneous impact of industrial robots on environmental pollution in different periods. The estimation results are shown in Table 13. Compared with 2006–2012, the emission reduction effect of industrial robots during 2013–2019 is greater.

**Table 13:** Regression results based on time heterogeneity.

	06–12			13–19		
	lnWW	lnSO_2_	lnSW	lnWW	lnSO_2_	lnSW
lnIR	− 0.182	− 0.207***	− 0.0109	− 0.207***	− 0.581***	− 0.543***
	(− 0.96)	(− 4.19)	(− 0.09)	(− 3.20)	(− 2.75)	(− 5.92)
lnGDP	− 0.0334	− 0.0453*	0.0170	− 0.547	− 2.258***	− 0.523**
	(− 0.98)	(− 1.74)	(0.35)	(− 1.31)	(− 4.86)	(− 2.02)
lnURB	0.398	− 0.218	− 0.643	− 2.329***	5.948***	0.788*
	(0.96)	(− 0.69)	(− 1.10)	(− 3.19)	(7.35)	(1.74)
lnEC	0.264**	− 0.0910	− 0.887***	− 0.167	0.0093	− 0.195
	(2.20)	(− 0.99)	(− 5.20)	(− 0.74)	(0.04)	(− 1.40)
lnGOV	0.0356	0.453***	1.144***	− 1.262***	− 1.032***	− 0.511**
	(0.20)	(3.32)	(4.51)	(− 3.70)	(− 2.72)	(− 2.41)
lnER	0.0736*	0.132***	0.0683	0.0342	0.0145	0.0269
	(1.91)	(4.52)	(1.25)	(0.57)	(0.22)	(0.73)
Constant	10.64***	5.051***	9.839***	27.96***	0.963	12.51***
	(6.40)	(3.99)	(4.17)	(4.70)	(0.15)	(3.39)
R^2^	0.1798	0.4626	0.7360	0.5544	0.9074	0.4206
N	210	210	210	210	210	210

*t* statistics in parentheses.

***p < 0.01, **p < 0.05, *p < 0.1.

## Discussion

The use of industrial robots can effectively reduce environmental pollution, which is consistent with hypothesis 1. This is contrary to the findings of Luan et al.^22^, who believed that the use of industrial robots would exacerbate air pollution. The inconsistency in research conclusions may be due to differences in research focus, sample size, and maturity of industrial robot technology. In terms of research focus, this paper mainly focuses on the role of industrial robots in reducing pollutant emissions during industrial production processes. Their research focuses more on the energy consumption caused by the production and use of industrial robots, which could aggravate environmental pollution. In terms of sample size, the sample size of this paper is 30 provinces in China from 2006 to 2019. These regions share consistency in economic development, industrial policies and environmental regulations. Their sample size is 74 countries from 1993 to 2019. These countries cover different geographical, economic and industrial development stages, affecting the combined effect of robots on environmental pollution. In terms of the maturity of industrial robots, the maturity of industrial robot technology has undergone tremendous changes from 1993 to 2019. In the early stages, industrial robot technology was immature, which might cause environmental pollution. In recent years, industrial robot technology has gradually matured, and its operating characteristics have become environmentally friendly. Their impact on environmental pollution has gradually improved. This paper mainly conducts research on the mature stage of industrial robot technology. Their research covers the transition period from immature to mature industrial robot technology. The primary reason that the use of industrial robots can reduce environmental pollution is: The use of industrial robots has a substitution effect on labor force, which reduces the raw materials needed for manual operation. For example, in the industrial spraying of manufacturing industry, the spraying robot can improve the spraying quality and material utilization rate, thereby reducing the waste of raw materials by manual operation. Zhang et al.^57^ argued that energy consumption has been the primary source of environmental pollution. Coal is the main energy in China, and the proportion of clean energy is low^58^. In 2022, clean energy such as natural gas, hydropower, wind power and solar power in China accounts for only 25.9% of the total energy consumption, which can cause serious environmental pollution problems. Industrial robots can promote the use of clean energy in industrial production and the upgrading of energy structure^24^. The reduction of raw materials and the upgrading of energy structure can control pollutant emissions in front-end production. On September 1, 2021, the World Economic Forum (WEF) released the report “*Using Artificial Intelligence to Accelerate Energy Transformation*”. The report points out that industrial robots can upgrade pollution monitoring equipment and sewage equipment, which reduces pollutant emissions in end-of-pipe treatment. Ye et al.^59^ also share the same viewpoint.

The use of industrial robots can reduce environmental pollution through green technology innovation, which is consistent with hypothesis 2. Industrial robots promote the integration of knowledge, which helps enterprises to carry out green technology innovation activities. Meanwhile, Jung et al.^60^ suggested that industrial robots can lower production costs for companies, allowing them to invest in green technology research. The level of green technology innovation is improved. Green technology innovation reduces environmental pollution through the following three aspects: Firstly, the improvement of energy utilization efficiency. China’s utilization efficiency of traditional energy sources such as coal is not high. The report of “*2013-Global Energy Industry Efficiency Research*” points out that China’s energy utilization rate is only ranked 74th in the world in 2013. Low energy efficiency brings serious environmental pollution problems^61^. Du et al.^62^ found that the innovation of green technologies, such as clean coal, can enhance energy efficiency and decrease environmental pollution. Secondly, the production of green products. Green technology innovation accelerates the green and recyclable process of production, thereby reducing the pollutants generated in production process. Thirdly, the improvement of enterprise competitive advantage. Green technology innovation can enable enterprises to gain greater competitive advantage in green development^63^. The supply of environmentally friendly products increases, which not only meets the green consumption needs of consumers, but also reduces the emission of pollutants.

Industrial robots can reduce environmental pollution by optimizing the structure of employment skills, which is consistent with hypothesis 3. Autor et al.^64^ contended that industrial robots would replace conventional manual labor positions, reducing the demand for low-skilled labor. Industrial robots represent the development of numerical intelligence. With the continuous development of digital intelligence, the demand for high-skilled labor in enterprises has increased. Koch et al.^65^ demonstrated that the use of industrial robots in Spanish manufacturing firms leads to an increase in the number of skilled workers. In February 2020, the Ministry of Human Resources and Social Security, the State Administration of Market Supervision and the National Bureau of Statistics jointly issues 16 new professions such as intelligent manufacturing engineering and technical personnel, industrial Internet engineering and technical personnel, and virtual reality engineering and technical personnel to the society. These new occupations increase the demand for highly skilled labor. The reduction of low-skilled labor and increase of high-skilled labor optimize the structure of employment skills. The optimization of employment skill structure narrows the wage gap between employees, reducing the communication cost of employees. Employees learn and exchange technology with each other, which not only improves the absorption capacity of clean technology. It also improves the production efficiency of enterprises and increases corporate profits, so that enterprises can use more funds for clean technology research and development, thereby reducing environmental pollution.

## Conclusions and policy recommendations

Based on the panel data of 30 provinces in China from 2006 to 2019, this paper uses the panel data model and mediating effect model to empirically test the impact of industrial robots on environmental pollution and its transmission mechanism. This paper uses panel quantile model, regional samples and time samples to further analyze the heterogeneous impact of industrial robots on environmental pollution. The conclusions are as follows: (1) Industrial robots can significantly reduce environmental pollution. For every 1% increase in industrial robots, the emissions of industrial wastewater, industrial SO_2_, and industrial dust and smoke decrease by − 0.242%, − 0.0875%, and − 0.277%. This finding is contrary to that of Luan et al.^22^, who argued that the use of industrial robots exacerbates air pollution. The results of this paper provide a contrasting perspective, highlighting the potential value of industrial robots in mitigating environmental pollution. (2) Industrial robots can reduce environmental pollution by improving green technology innovation level and optimizing employment skills structure. In the impact of industrial robots on industrial wastewater discharge, the mediating effect of green technology innovation accounts for 8.17% of total effect. The mediating effect of employment skill structure accounts for 6.67% of total effect. In the impact of industrial robots on industrial SO_2_ emissions, the mediating effect of green technology innovation accounts for 11.8% of total effect. The mediating effect of employment skill structure accounts for 20.66% of total effect. In the impact of industrial robots on industrial soot emissions, the mediating effect of green technology innovation accounts for 3.72% of total effect. The mediating effect of employment skill structure accounts for 15.53% of total effect. While Obobisa et al.^66^ and Zhang et al.^67^ highlighted the role of green technological innovation in addressing environmental pollution. Chiacchio et al.^68^ and Dekle^69^ focused on the effects of industrial robots on employment. The mediating impact of technology and employment in the context of robots affecting pollution hasn’t been addressed. Our research provides the first in-depth exploration of this crucial intersection. (3) Under different environmental pollution levels, the impact of industrial robots on environmental pollution is different. Among them, with the increase of industrial wastewater discharge, the impact of industrial robots on industrial wastewater discharge shows a “W-shaped” change. With the increase of industrial SO_2_ emissions, the negative impact of industrial robots on industrial SO_2_ emissions is gradually increasing. On the contrary, with the increase of industrial soot emissions, the negative impact of industrial robots on industrial soot emissions gradually weakens. (4) Industrial robots in different regions and different periods have heterogeneous effects on environmental pollution. Regarding regional heterogeneity, industrial robots in eastern region have the greatest negative impact on environmental pollution, followed by central region, and western region has the least negative impact on environmental pollution. Regarding time heterogeneity, the negative impact of industrial robots on environmental pollution in 2013–2019 is greater than that in 2006–2012. Chen et al.^5^ and Li et al.^24^ both examined the overarching impact of industrial robots on environmental pollution. They did not consider the varying effects of robots on pollution across different regions and time periods. Breaking away from the limitations of previous holistic approaches, our study offers scholars a deeper understanding of the diverse environmental effects of industrial robots.

According to the above research conclusions, this paper believes that the government and enterprises can promote emission reduction through industrial robots from the following aspects.

1. Increase the scale of investment in robot industry and promote the development of robot industry. China’s industrial robot ownership ranks first in the world. Its industrial robot installation density is lower than that of developed countries such as the United States, Japan and South Korea. The Chinese government should give some financial support to robot industry and promote the development of robot industry, so as to effectively reduce environmental pollution. The R&D investment of industrial robots should be increased so that they can play a full role in reducing raw material consumption, improving energy efficiency and sewage treatment capacity.
2. Give full play to the role of industrial robots in promoting green technology innovation. Industrial robots can reduce environmental pollution through green technology innovation. The role of industrial robots in innovation should be highly valued. The advantages of knowledge integration and data processing of industrial robots should be fully utilized. Meanwhile, the government should support high-polluting enterprises that do not have industrial robots from the aspects of capital, talents and technology, so as to open up the channels for these enterprises to develop and improve clean technology by using industrial robots.
3. Give full play to the role of industrial robots in optimizing employment skills structure. The use of industrial robots can create jobs with higher skill requirements and increase the demand for highly skilled talents. China is relatively short of talents in the field of emerging technologies. The education department should actively build disciplines related to industrial robots to provide talent support for high-skilled positions. Enterprises can also improve the skill level of the existing labor force through on-the-job training and job competition.

### Supplementary Information

Supplementary Information.

## Data Availability

The datasets used or analyzed during the current study are available from Yanfang Liu on reasonable request.
