# Supplementary material for: Impact of industrial robots on environmental pollution: evidence from China
Source: Sci Rep. 2023 Nov 26;13:20769. doi: 10.1038/s41598-023-47380-6 (PMC10679152; doi:10.1038/s41598-023-47380-6)
Supplement: Supplementary file 1 — Supplementary Information. [file 41598_2023_47380_MOESM1_ESM.docx]

**Appendix A**

Coding Results of the Main Articles Included in the Meta-Analysis

| Subject | Research object | Sample size | Explanatory variables | Correlation coefficient |
| --- | --- | --- | --- | --- |
| 1 How does green investment affect environmental pollution?  Evidence from China | 30 provinces in China | 360 | Green investment | -0.051 |
| 2 Does foreign direct investment affect environmental pollution in China's cities? 3 A spatial econometric perspective | 285 cities in China | 3440 | Foreign direct investment | -0.03、0.009、0.003 |
| Potential heterogeneity in the relationship between urbanization and air pollution, from the perspective of urban agglomeration | 13 regions of Beijing, Tianjin and Hebei | 65 | Urbanization | 0.097 |
| 4 The influence of increased population density in China on air pollution | 284 cities in China | 3976 | Population density | -0.206 |
| 5 The impact of technological innovation on air pollution: Firm-level evidence from China | 11625 companies in China | 88680 | Technological innovation | -0.0271 |
| 6 Effects of heterogeneous technological progress on haze pollution: Evidence from China | 30 provinces in China | 420 | Technological progress | -0.241 |
| 7 Can the new energy demonstration city policy reduce environmental pollution? Evidence from a quasi-natural experiment in China | 271 cities in China | 3252 | New energy demonstration city policy | -0.2883、-0.1283 |
| 8 How does the use of industrial robots affect the ecological footprint? International evidence | 76 countries | 1944 | Industrial robots | 0.201 |
